# Angiotensin II, dopamine and nitric oxide. An asymmetrical neurovisceral interaction between brain and plasma to regulate blood pressure

**DOI:** 10.3934/Neuroscience.2019.3.116

**Published:** 2019-07-26

**Authors:** I. Banegas, I. Prieto, A.B. Segarra, M. Martínez-Cañamero, M. de Gasparo, M. Ramírez-Sánchez

**Affiliations:** 1 Department of Health Sciences, University of Jaén, Jaén, Spain; 2 Cardiovascular and Metabolic Syndrome Adviser, Rossemaison, Switzerland

**Keywords:** angiotensin, dopamine, nitric oxide, brain asymmetry, aminopeptidases, blood pressure

## Abstract

Vital functions, such as blood pressure, are regulated within a framework of neurovisceral integration in which various factors are involved under normal conditions maintaining a delicate balance. Imbalance of any of these factors can lead to various pathologies. Blood pressure control is the result of the balanced action of central and peripheral factors that increase or decrease. Special attention for blood pressure control was put on the neurovisceral interaction between Angiotensin II and the enzymes that regulate its activity as well as on nitric oxide and dopamine. Several studies have shown that such interaction is asymmetrically organized. These studies suggest that the neuronal activity related to the production of nitric oxide in plasma is also lateralized and, consequently, changes in plasma nitric oxide influence neuronal function. This observation provides a new aspect revealing the complexity of the blood pressure regulation and, undoubtedly, makes such study more motivating as it may affect the approach for treatment.

## 1. Introduction

It is clearly accepted that the regulation of vital functions such as blood pressure is carried out through the equilibrium between various factors, sometimes with opposite functions, such as those that increase or decrease cardiac output, blood volume or peripheral resistance [1]. In the same way, for the control of blood pressure, a correct interaction between central and peripheral mechanisms is involved [2]–[4]. Furthermore, complex interactions between environmental extrinsic factors and behavioral ones and intrinsic mechanisms of cardiovascular regulation are implicated [5]. In addition, the recent interest in the relation of the brain and peripheral asymmetric organization of the nervous system for the control of blood pressure is increasing [3],[6]–[10]. Among the factors that interact at all these levels, the role of the renin-angiotensin aldosterone system, dopamine and nitric oxide is highlighted [7]. The present article analyzes the most significant reports indicating how these factors interact from a bilateral point of view and provides new relevant data for the understanding of this complex system.

## 2. Renin-Angiotensin aldosterone system

**Figure 1.**
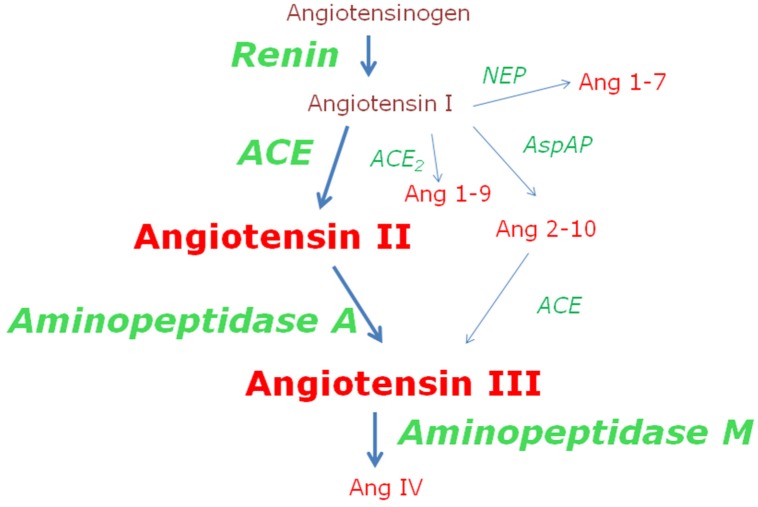
The renin-angiotensin aldosterone system. Simplified scheme of the RAAS in which its most active components (peptides and enzymes) stand out in bold with a larger size. In brown, peptides without known biological activity. In red, some of the peptides for which various functions have been described. In green, some of the enzymes responsible for the formation and inactivation of the various angiotensins. The formation of angiotensin II, by the action of ACE on angiotensin I, and its subsequent hydrolysis by aminopeptidase A, leading to the formation of angiotensin III, which will be subsequently inactivated by aminopeptidase M, are important steps especially highlighted in the figure. Angiotensin II is classically considered as the main peptide of the system, target of various therapeutic strategies (inhibiting its formation or blocking its action) for the treatment of hypertension [12]. Angiotensin III is believed to be the main angiotensin in brain, with hypertensive properties. Recent therapeutic strategies against hypertension are aimed at reducing its formation through the use of central inhibitors of aminopeptidase A [12]–[15] From the dynamics of the enzymatic cascade, low levels of substrate can be deduced from high levels of the enzyme that hydrolyzes it and vice versa. ACE, angiotensin converting enzyme; AspAP, aspartate aminopeptidase; NEP, neutral endopeptidase.

The role of the renin-angiotensin aldosterone system (RAAS) is essential in the control of blood pressure. In fact, most of the anti-hypertensive treatment's protocols include drugs that inhibit or block the RAAS [11] at various levels such as the peripheral inhibition of renin or angiotensin-converting enzyme (ACE), the central inhibition of aminopeptidase A or the blockade of the angiotensin (Ang) II receptor [12]. In the RAAS, the function of the active peptides of the cascade depends on the activity of the enzymes controlling the formation or inactivation of these peptides and not on a RNA-dependent mechanism. Only angiotensinogen, an inactive peptide precursor of the cascade, requires such RNA-dependent mechanism. Therefore, the complex regulation of the formation and inactivation of the various angiotensins, some of them with opposite functions, is conditioned by a subtle coordination between the activities of the enzymes that make up the system (Figure 1) [12]–[15].

The function of brain angiotensin-hydrolyzing aminopeptidases in hypertension has been studied by various authors. Wright et al. [16] showed that after the intracerebroventricular (icv) injection of Ang II and Ang III in spontaneously hypertensive rats (SHR), more sensitivity and a more prolonged increase in blood pressure were observed than in normotensive Wistar-Kyoto rats (WKY). Further, pretreatment with bestatin (an aminopeptidase M inhibitor) preventing the inactivation Ang III potentiated and prolonged the increase of blood pressure after the injection of Ang II and Ang III in SHR. Wright et al. [17] also demonstrated that the icv injection of aminopeptidase M reduced significantly blood pressure in both WKY and SHR due to the central inactivation of Ang III. Llorens-Cortes and her research team also analyzed the role of cerebral aminopeptidase A and M in the control of blood pressure and hydro-electrolytic balance. They demonstrated that the action of hypothalamic Ang II on the release of vasopressin depends on the previous biotransformation of Ang II to Ang III by the action of aminopeptidase A [18]. The subsequent inhibition of aminopeptidase M induces an increase in the release of vasopressin because it enhances and prolongs the action of Ang III. A promising therapeutic strategy in the treatment of hypertension may consist in the development of new drugs aimed at reducing the formation of Ang III through the inhibition of aminopeptidase A [13]–[15].

Therefore, a key role in the central and peripheral function of the RAAS is linked to aminopeptidase A (EC 3.4.11.7) and aminopeptidase M (EC 3.4.11.2) respectively responsible for inactivating angiotensin II and promoting the formation of angiotensin III, and for inactivating angiotensin III [12].

## 3. Dopamine

**Figure 2.**
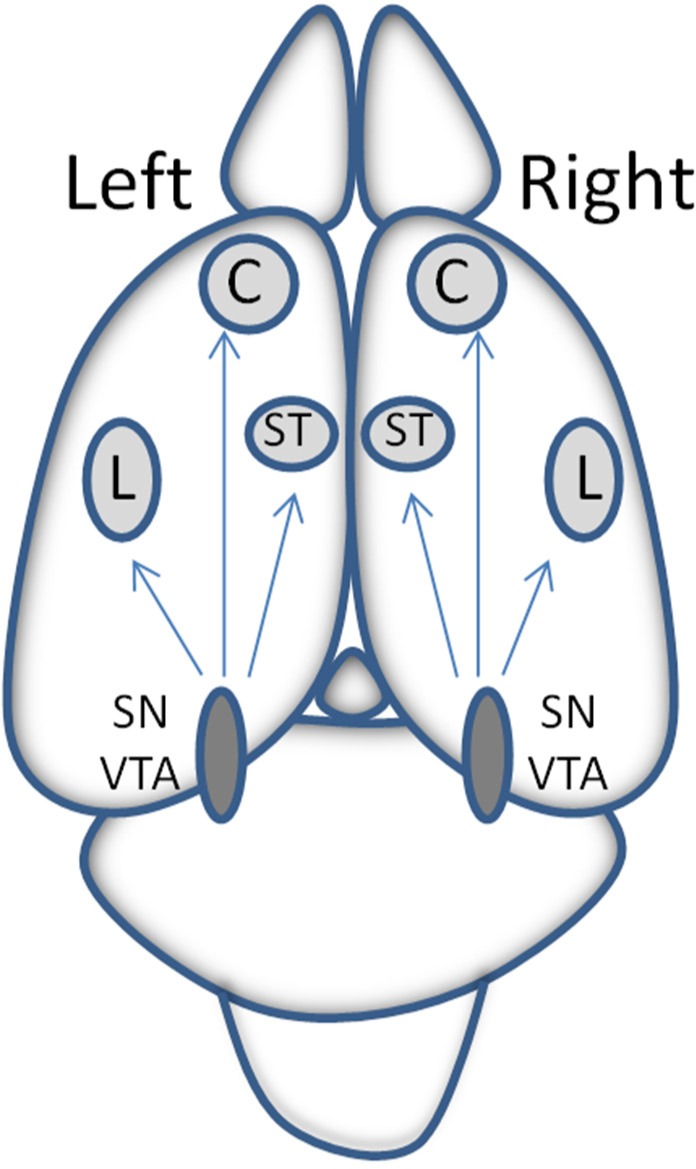
Brain dopaminergic system. Simplified representation of the brain dopaminergic system. From the substantia nigra (SN) and ventrotegmental area (VTA) the dopaminergic neurons project unilaterally, without bilateral crossing, toward the cortex (C), limbic system (L) or striatum (ST). This distribution allows to selectively study the consequences of unilateral depletions of dopamine from the left or right hemisphere, offering a valuable tool for the study of the function of brain asymmetry [19].

The neuronal bodies, from which all the cerebral dopamine originates, are located in the brainstem, mainly in the pars compact of the substantia nigra and in the ventrotegmental area. Its brain distribution is unilateral without bilateral crossing [19] (Figure 2).

The relationship between dopamine and the control of blood pressure has been known for quite a while: whereas its central administration decreased blood pressure, the intravenous injection increased it [20]. The hypertensive effect of peripheral dopamine administration was abolished by treatment with the alpha-adrenergic receptor blocker phentolamine. In contrast, the decrease in blood pressure induced by the central administration of dopamine was not affected by pre-treatment with phentolamine but it was blocked by metoclopramide, an antagonist of the central D2 receptors. Further, central administration of metoclopramide decreases blood pressure suggesting that central D2 receptors, more than peripheral alpha adrenergic receptors, are involved in the control of blood pressure. The same authors proposed that the regulatory role of the brain dopaminergic system on blood pressure is linked to changes in the RAAS [20]. More recent studies suggest that, in basal conditions, dopamine and its receptors acting through a natriuretic effect counteract the hypertensive effect of the stimulation of alpha-adrenergic receptors and the action of the hypertensive components of the RAAS at the peripheral level. An abnormal interaction of such factors could be involved in the pathogenesis of hypertension [21].

## 4. Nitric oxide

Nitric oxide is a gas, highly diffusible through cell membranes, which produces vasodilatation and therefore is of hypotensive nature. It also acts as a neurotransmitter with important functions such as synaptic plasticity or memory processes. It is therefore an important central and peripheral regulator of the blood pressure [22]. It is produced by the action of various nitric oxide synthases (NOS) (EC 1.14.13.39): endothelial (eNOS), inducible (iNOS) and neuronal (nNOS). Due to the impossibility of limiting its diffusion between compartments, the nitric oxide produced by the action of eNOS or iNOS will be able to diffuse towards the nervous system: alterations in its plasma levels may produce not only alterations of its peripheral functions related to the vascular tone [23], but also contribute to alter the normal interaction exerted by the central nitric oxide with other blood pressure regulating factors, changing the central vascular function [24] and/or participating in the pathogenesis of various neurodegenerative diseases [25], schizophrenia and other psychiatric disorders such as bipolar disorders or depression [26]. For example, the decrease in the availability of nitric oxide has been related to its cortical and peripheral reduction that occurs during aging and with the severity of Alzheimer's disease [24]. Reduced levels of nitric oxide in plasma have also been associated with the pathophysiology of negative symptoms in schizophrenia [27].

Although nNOS has been studied primarily in brain, it is also found peripherally in perivascular nerves, heart or skeletal muscle. Therefore, although the importance of eNOS in cardiovascular regulation is clearly established, the participation of nNOS in the physiological regulation of systemic vascular resistance and blood pressure in healthy humans by modulating sympathetic activity, renal release of renin, hydro-electrolytic balance as well as cardiac function is also proposed [28].

Ischemic or hemorrhagic stroke is a serious acute cerebrovascular process in which nitric oxide could play an important role due to its essential role in the maintenance of vascular tone. It has been suggested that nitric oxide derived from the action of iNOS and nNOS have neurotoxic character while nitric oxide derived from the action of eNOS have a neuroprotective role in acute ischemic stroke. In relation to cerebral hemorrhage, there is no clear differentiation in the role that nitric oxide derived from the different NOS isoenzymes can exert, suggesting that an increase in nitric oxide levels after cerebral hemorrhage would improve the prognosis of these patients [29]. This has been hypothesized on the basis of plasma studies in animal models with subarachnoid hemorrhage [30] and in patients after acute stroke [31]. More recently, data supporting a relationship of eNOS with the cognitive alteration that occurs after an acute ischemic stroke have been provided [32].

## 5. Lateralization in the neurovisceral integration

The concept of “*Neurovisceral Integration*” in the control of blood pressure is more than 100 years old [33],[34]. However, diverse data are recently accumulating which extend the idea that the bidirectional interaction between brain and peripheral tissues for the control of blood pressure is asymmetrically organized [3].

We have contributed to studies that suggest a bidirectional connection between the brain and various peripheral tissues, such as heart and kidney, for the control of blood pressure and hydro-electrolytic balance: these involve several enzymes of the RAAS cascade. Such neuro-visceral interaction is modified by changes in blood pressure induced by the use of vasoactive hypertensive or hypotensive drugs and suggests to be mediated by the autonomic nervous system [2].

On the other hand, changes in blood pressure induce variations in the bilateral brain distribution of aminopeptidase activities as well as in the intra-hemispheric and inter-hemispheric interactions established between several of these enzymes [10]. While in normotensive animals, and in those animals that were treated with the hypotensive drug captopril, appeared a predominance of intra-hemispheric correlations into the left hemisphere, the hypertensive animals and those treated with the hypertensive drug L-NAME exhibited it into the right one. An asymmetry was also observed in the inter-hemispheric correlations that were established between aminopeptidase activities of the left and right hemisphere: there were more inter-hemispheric correlations in normotensive animals and in those treated with antihypertensive drugs than in hypertensive patients or those treated with hypertensive drugs [10].

Finally, other data suggest that this neurovisceral interaction between aminopeptidases that hydrolyze angiotensin peptides in the control of blood pressure is asymmetrically organized. An asymmetry has been observed in the correlation established between the frontal cortex and the plasma in hypertensive animals: while in hypertensive animals a correlation between plasma and the right frontal cortex is observed, such correlation is established with the left frontal cortex after the treatment with captopril and the related reduction of blood pressure [35]. The relationship established between the frontal cortex and the cardiac tissue, specifically the left ventricle, is also asymmetric being the opposite in comparison with the correlation observed with plasma: the most significant correlation between frontal cortex and ventricle was observed with the right frontal cortex in the animals treated with captopril compared with the control group of hypertensive animals [36].

All these previously reviewed factors, renin-angiotensin aldosterone system, dopamine, nitric oxide and asymmetry in neurovisceral integration for the control of blood pressure could be inter-connected.

## 6. Asymmetry in the neurovisceral interaction between nitric oxide, dopamine and renin-angiotensin aldosterone system

**Figure 3.**
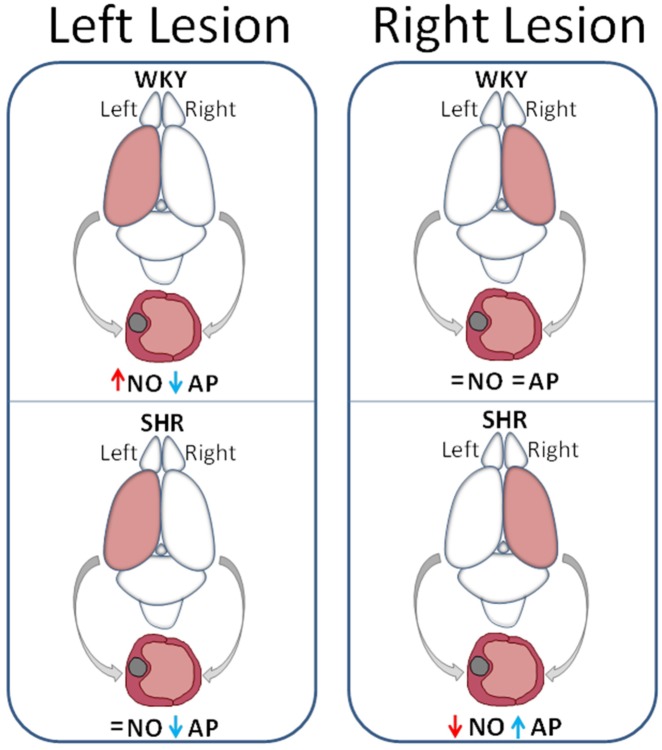
Plasma nitric oxide and aminopeptidase activity after left or right lesion. Schematic representation of the consequences of left or right nigrostriatal lesion on plasma levels of nitric oxide (NO) (red arrows) and aminopeptidase A (AP) (blue arrows) in normotensive animals (WKY) and spontaneous hypertensive rats (SHR). (=) indicates that there is no variation compared to control animals. The lesioned hemisphere in the rat brain appears in brown color. Drawings under brains represent blood vessels with sectioned endothelial cells. Gray arrows represent the left or right autonomic innervation of the blood vessels [7].

The asymmetric behavior of the relationship between nitric oxide, dopamine and RAAS in blood pressure control was not apparent until the work of Banegas et al. [7]. The unilateral distribution of dopamine, without inter-hemispheric crossing, offers us a valuable tool to selectively analyze the response of one or the other side depending on whether we eliminate the dopamine from the left or the right hemisphere [19]. The results demonstrated that blood pressure levels as well as aminopeptidase activities in brain and plasma and nitric oxide in plasma varied depending on whether the animals were normotensive or hypertensive and whether the injured hemisphere (to deplete dopamine) was the left or the right one. The interaction between factors also varied depending on whether the lesion was performed on one side of the brain or the other [7]–[9] (Figure 3).

The asymmetric character of Parkinson's disease produced by a lateralized distribution of brain dopamine is not only manifested by lateralized consequences of motor behavior but also by the rise of cognitive and autonomic changes including cardiovascular alterations [37] in which the nitric oxide may have a relevant role [38].

Banegas et al. [8] showed a marked increase in blood pressure in animals in which dopamine had been depleted from the left hemisphere following left unilateral injection of the neurotoxic 6-hydroxydopamine in comparison with the right side lesions that did not induce variations in blood pressure or these were much less pronounced. Because heart rate in these animals did not change after lesion, the authors suggested that the marked increase of blood pressure in the animals with left lesions was due to an increase in the peripheral resistances rather than an increase in cardiac output, a mechanism linked to an asymmetry in the vascular autonomic control. Alternatively, since an asymmetrical baroreceptor activity was reported, possibly due in part to a lateralization of tyroxine hydroxylase activity in the median eminence [39], the left or right depletion of dopamine could influence differentially on blood pressure blunting asymmetrically the baroreceptor activity.

The asymmetric behavior after the left or right unilateral lesions of the nigrostriatal system is not only reflected in changes in blood pressure but also extends to differential changes in the levels of plasma factors which are depending on whether the lesion occurred on the left side or in the right one. Among others, nitric oxide and aminopeptidase A are modified in plasma. While in normotensive animals (WKY) the left lesion increases plasma nitric oxide and reduces aminopeptidase A, the right lesion does not modify either of the two factors. In contrast, in hypertensive animals (SHR) while the left lesion does not modify nitric oxide and decreases aminopepidase A, the right lesion decreases nitric oxide and increases aminopeptidase A. This is just the inverse behavior to what happened in normotensive animals with left lesion (Figure 3). If we take into account the ratio “high enzymatic activity/low level of hydrolyzed substrate” and vice versa (Figure 1), we can suggest that the left lesion maintains elevated plasma levels of angiotensin II in WKY and SHR whereas the right lesion does not modify (in WKY) or decreases them (in SHR).

The correlation study between nitric oxide and plasma aminopeptidase A in the various groups studied showed significant negative correlations (the higher nitric oxide level, the lower aminopeptidase A and vice versa) between aminopeptidase A and nitric oxide in the WKY group with right lesion and in the group of SHR with left lesion.

**Figure 4.**
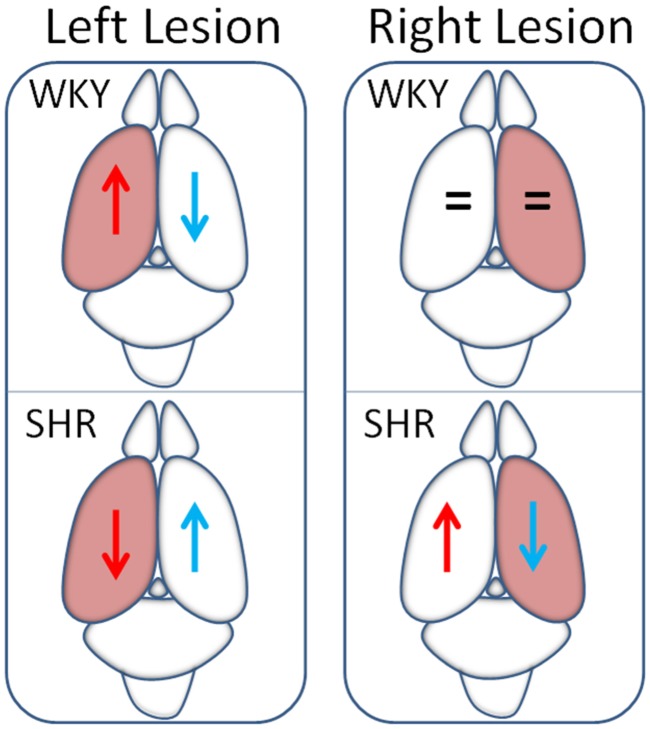
Hypothetic Ang III levels after left or right nigrostriatal lesions. Schematic representation of the hypothetical levels of Ang III in the left (red arrows) or right (blue arrows) hemisphere, considering the results obtained for aminopeptidase M activity (high enzymatic activity /low Ang III concentration and vice versa) in groups of WKY or SHR in which the left or right nigrostriatal systems were lesioned [9].

Banegas et al. [9] described the bilateral behavior of aminopeptidase M activity and its role as enkephalinase in rats with left or right lesions of the nigrostriatal system [40]. However, this enzyme also hydrolyzes angiotensin III [41] (Figure 1). It is therefore interesting to analyze these results considering this other enzymatic target. It has been demonstrated that angiotensin III is the most active peptide of the RAAS in the brain and its cerebral function is clearly hypertensive [41]. In comparison with control animals, left side-lesioned WKY animals showed a decrease in aminopeptidase M in the left hemisphere and an increase in the right one. In SHR, the left lesion produces exactly the opposite: an increase in the left hemisphere and a decrease in the right one. The right lesion in WKY did not modify the activity of aminopeptidase M in comparison with the control animals. In contrast, in the SHR this activity decreased in the left side and increased in the right one (Figure 4).

If we consider the relationship “high enzymatic activity/low concentration of the substrate” and vice versa, we would obtain angiotensin III levels that would correspond to the inverse behavior of the previously described one for aminopeptidase M i.e. in the left lesion WKY animals, the left hemisphere would have elevated levels of angiotensin III and the right one decreased values compared with control animals. In comparison with the WKY, the left lesion in the SHR would result in an opposite response: low levels of Angiotensin III in the left side and high levels in the right one. Also, the right lesion in SHR increases Ang III in the left side but decreases it in the right one (Figure 4). This is in contrast with WKY where the right lesion does not modify the aminopeptidase M (and consequently Ang III) in any of the hemispheres compared to controls.

## 7. Concluding remarks

Physiology is an integrating discipline, in which it is clearly shown that there are no independent compartments in the functioning of the organism but it works as an integrated whole. The control of cardiovascular function is an example of this integration. In this review we have discussed how factors with very diverse functions and from several locations can interact with each other for the control of blood pressure. From our results, it is also deduced that the neurovisceral integration for the control of blood pressure is organized asymmetrically and the response is not the same if the alteration occur on the left or right side of the brain. In relation to cardiovascular function, we could hypothesize from these results a greater severity in the processes that affect the left hemisphere than in those involving the right one. This situation would have to be taken into account in processes such as for example Parkinson's disease or schizophrenia in which an asymmetric development and evolution of the disease is clearly demonstrated. The vascular and/or autonomic consequences will vary depending on the alteration of the nitric oxide if the process involves one side or the other of the brain. Indeed, in brain strokes involving the left or right hemisphere, we have been able to confirm that the response in the plasma levels of nitric oxide varies depending on the injured side. A possible beneficial treatment aimed to restore nitric oxide levels will therefore depend on the affected brain side. The understanding of the bilateral behavior of the interaction of multiple factors with functions apparently opposite in some cases constitutes a complex challenge. Thus further studies are necessary to structure the bilateral behavior of the nervous system and the peripheral response in the control of blood pressure.
